# Tea (*Camellia sinensis*) Extract-Mediated Green Synthesis of Co_3_O_4_ and Co_3_O_4_@Graphene Nanocomposites for Multifunctional Applications in Pollutant Degradation, Sensing, and Energy Storage

**DOI:** 10.1021/acs.langmuir.5c04204

**Published:** 2026-02-16

**Authors:** Lakshmanan Ganesh, Sundararajan Ashok Kumar, Balamurugan Arumugam, Mariadhas Jarvin, Daniel Rani Rosaline, Nelson Y. Dzade, Savariroyan Stephen Rajkumar Inbanathan

**Affiliations:** † Post Graduate and Research Department of Physics, 563171 The American College, Madurai, Tamil Nadu 625002, India; ‡ Department of Physics, 250161 Sree Sakthi Engineering College, Karamadai, Coimbatore, Tamil Nadu 641104, India; § PG and Research Department of Chemistry, 29967 Thiagarajar College, Madurai, Tamil Nadu 625009, India; ∥ Department of Chemistry, National Sun Yat-Sen University, Kaohsiung 80424, Taiwan; ⊥ Post Graduate and Research Department of Chemistry, Lady Doak College, Madurai, Tamil Nadu 625002, India; # Department of Energy and Mineral Engineering, 311285 The Pennsylvania State University, University Park, Pennsylvania 16802, United States

## Abstract

A novel solution-mixing method was proposed to synthesize Co_3_O_4_/graphene nanocomposites (Co_3_O_4_@Gr) using a green tea leaf (*Camellia sinensis*) extract as the reducing agent. XRD analysis shows that the as-prepared Co_3_O_4_@Gr exhibits a cubic spinel crystal structure. From morphological analysis, the obtained Co_3_O_4_ NS forms spherical clusters that are uniformly distributed on the graphene surface. FT-IR and Raman analyses confirmed the strong molecular and vibrational interactions between the Co_3_O_4_ NS and Gr. The suppressed PL intensity peak of the Co_3_O_4_@Gr NCs indicated significant inhibition in the recombination of charge carriers between the hybrid orbitals within the composites. As a result, the catalytic efficiency of Co_3_O_4_@Gr NCs increased to 80% compared to pristine Co_3_O_4_, which exhibited only 45% efficiency against methylene blue (MB) dye. Moreover, the as-prepared NCs exhibited a detection limit of 0.01–224 μM, demonstrating a superior low-DPA detection with high sensitivity. The Co_3_O_4_@Gr/GCE exhibits admirable selectivity for various pesticides, fungicides, and metal ions, with outstanding reproducibility and stability. From electrochemical investigations, the highest specific capacitance values of the as-synthesized Co_3_O_4_@Gr were 349 F/g at a scan rate of 5 mV/s and 158 F/g at a current density of 1 A/g.

## Graphical abstract

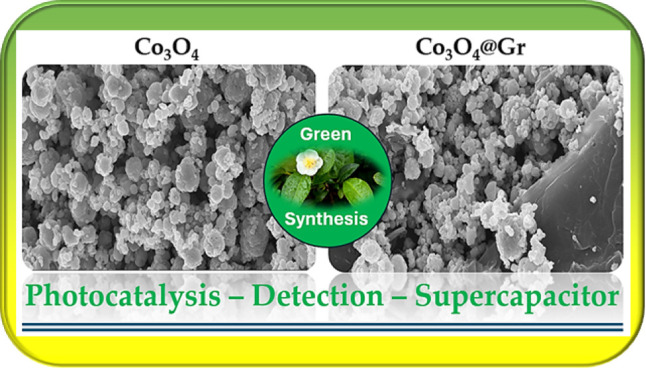

## Introduction

In recent years, researchers have become increasingly interested in transition-metal oxides and doped metal-oxide/graphene nanocomposites (NCs) for multipurpose applications. Metal oxides, such as NiO, ZnO, MnO_2_, WO_3_, RuO_2_, and Co_3_O_4_,^1–3^ have been explored for supercapacitor applications due to their high theoretical capacitance. Among these, Co_3_O_4_ is a suitable material for supercapacitor applications owing to its high theoretical capacitance, high surface-to-volume ratio, high chemical stability, and outstanding electrochemical redox activity.^4,5^ Co_3_O_4_ is crystallized in the spinel cubic structure with Co^2+^ ions (magnetic) in the tetragonal site and Co^3+^ ions (nonmagnetic) in the octahedral site.^6^ The structure of Co_3_O_4_ plays a significant role in its supercapacitor application. Based on the structure, the charge-transfer kinetics at the electrode/electrolyte interface vary. However, the low electrical conductivity of Co_3_O_4_ severely limits its potential for energy conversion and storage applications. By synthesizing Co_3_O_4_ nanoparticles in various morphologies, such as porous, nanowire, nanosphere, nanoflower, nanorod, and nanoplate,^7–10^ the supercapacitor performance can be enhanced. Common synthetic methods for producing metal oxide nanoparticles include the reflux method, the sol–gel method, and hydrothermal decomposition. However, these methods require high operating temperatures and challenging experimental setups and may be less practical for mass production.^11,12^ Green synthesis offers an attractive method for large-scale synthesis of NS at lower reaction temperatures.^13–16^

The need for renewable and sustainable energy is driving a higher demand for energy storage technologies. Portable electronic devices and vehicles require high efficiency, low cost, good stability, and an ecofriendly power source. On this basis, supercapacitors (ultracapacitors or electrochemical capacitors) have attracted significant attention due to their high energy-storage capacity.^17,18^ Supercapacitors can be classified into three different types based on their charge storage mechanism:^19–22^ (i) electric double-layer capacitor (EDLC), (ii) pseudocapacitor, (iii) hybrid capacitor. EDLC is the charge stored at the electrode–electrolyte interface (capacitive). Carbon-based materials such as GO, rGO, and activated carbon exhibit an EDLC behavior. In a pseudocapacitor, a redox reaction occurs at the electrode–electrolyte interface and ions are absorbed by the electrode via a surface reaction. Noble transition metal oxides or conducting polymers are generally used as electrode material for pseudocapacitors.^23–26^ The hybrid capacitor is a combination of EDLC and pseudocapacitor, so it possesses both capacitive (EDLC) and redox (pseudocapacitor) behavior.^27–31^

Enhancing large-scale water treatment processes has historically relied on strategies that address challenges such as agglomeration, inactivation, operational complexity, and environmental risks. One highly effective approach involves leveraging nanomaterials with fixed confined structures. Transition-metal oxide NSs have emerged as a solution for the degradation of organic contaminants in polluted water. Dye effluents from textile industries are harmful to our environment, especially azo dyes, which can cause cancer and threaten both humans and aquatic systems. Methylene blue (MB) dye is used mainly in textiles and is toxic to living beings. Traditional wastewater treatment methods often fail to remove these dyes because they are stable and complex. One effective method is photodegradation, which uses UV or visible light to degrade the toxic dye. Many studies have examined semiconductor-based photocatalysts for removing these organic molecules from wastewater, as they can be tailored to meet different needs.^32–35^ Notably, nanostructured Co_3_O_4_ nanoparticles (NS) exhibit remarkable photocatalytic activity against various toxic dyes. This enhanced performance can be attributed to their significantly high surface-to-volume ratio, which results in an abundance of active surface sites. Graphene (Gr) represents a prime example of such confined 2D carbon materials, which when combined with Co_3_O_4_ NS, offers a high specific surface area. Previous studies have demonstrated that graphene suppresses the electron–hole pair recombination (EHP) and increases the degradation efficiency.^36–41^

Diphenylamine (DPA) is an insecticide and an organic derivative of aniline with the chemical formula C_12_H_11_N. It has mainly been used as a postharvest scald inhibitor for apples and pears because of its strong antioxidant properties, which help preserve fruit skin by preventing oxidative breakdown and degradation of α-farnesene, a naturally occurring surface compound. DPA has low solubility but can adhere to fruit surfaces and may persist in processed products such as fruit juices. It can also be used industrially in pharmaceutical production, in rubber, plastics, dyes, and photographic chemicals, and as a stabilizer in propellants. Nevertheless, DPA and its derivatives are present in wastewater from storage facilities and are associated with adverse health outcomes, including damage to red blood cells, bladder problems, and dermatological conditions. The European Union classifies DPA as a significant pollutant. Conventional methods for DPA detection include gas chromatography, HPLC, mass spectrometry, and spectrophotometry.^42,43^ Electrochemical detection is also a promising alternative. Many studies have focused on DPA measurements using metal oxides, such as CuO, NiO, and Co_3_O_4_. Among these, Co_3_O_4_ stands out due to its electrocatalytic properties and robust chemical stability. The Co_3_O_4_-based DPA sensor, in particular, has garnered significant attention for its outstanding performance in electrooxidizing DPA, particularly in alkaline solutions.^44,45^

In this study, we used a novel microwave-assisted solution-mixing method to synthesize Co_3_O_4_/graphene nanocomposites (Co_3_O_4_@Gr) using green tea leaf extract as the reducing agent. The phase, crystallinity, and morphology of the as-prepared Co_3_O_4_@Gr nanocomposites were characterized, and their catalytic activity toward (MB) dye degradation, electrochemical detection of dopamine (DPA), and electrochemical specific capacitance were analyzed. The Co_3_O_4_@Gr NCs exhibited enhanced efficiency (80%) in MB dye degradation and a superior low DPA detection limit (0.01–224 μM) with high sensitivity. The as-synthesized Co_3_O_4_@Gr also showed high specific capacitance values of 349 F/g at a scan rate of 5 mV/s and 158 F/g at a current density of 1 A/g.

## Experimental Procedures

### Preparation of Tea Extract

The tea bags (*Camellia sinensis* *) were purchased from a standard* commercial tea vendor (Brand: Taj Mahal, India). The tea extract was prepared by dipping a tea bag (5 g) into 100 mL of DI water and boiling it at 100 °C for 20 min. The obtained thick, brown tea extract was filtered through Whatman filter paper in a cleaned beaker and stored in a refrigerator for further use.

### Synthesis of Spinel Cobalt Oxide Nanosphere (NS)

The starting material, Co­(No_3_)_2_·6H_2_O (99.0% purity), was purchased from Isochem laboratories kochi. Co_3_O_4_ was synthesized by using microwave-assisted methods. Briefly, 2.5 g of Co­(No_3_)_2_·6H_2_O was dissolved in 100 mL of double deionized water (DD water) and stirred continuously for 1 h on a magnetic stirrer at room temperature. Subsequently, 50 mL of the *Camellia sinensis* tea extract was added dropwise to the solution over 1 h. The resulting solution was then transferred to a Petri dish, dried, and precipitated on a hot plate at 80 °C. The precipitate was ground in an agate mortar for 1 h to obtain a fine powder and then transferred to a crucible for calcination at 600 °C for 2 h.

The formation of Co_3_O_4_ nanoparticles from cobalt nitrate in the presence of green tea extract proceeds via a bioreduction-controlled oxidation mechanism mediated by plant phytochemicals. Initially, Co^2+^ ions from Co­(NO_3_)_2_·6H_2_O form coordination complexes with phenolic −OH and carbonyl groups of the phytochemicals. The polyphenols are oxidized to their corresponding quinones, simultaneously reducing a fraction of the Co^2+^ species to lower oxidation intermediates, such as Co­(OH)_2_. The hydroxide intermediates aggregate into nanoclusters, with the biomolecules serving as capping agents that restrict particle growth and prevent agglomeration. Subsequent thermal treatment (600 °C) under air atmosphere conditions converts Co­(OH)_2_ into spinel Co_3_O_4_. The detailed schematic of the synthesis procedure of spinel Co_3_O_4_ NS is illustrated in Figure 1.

**Figure 1:**
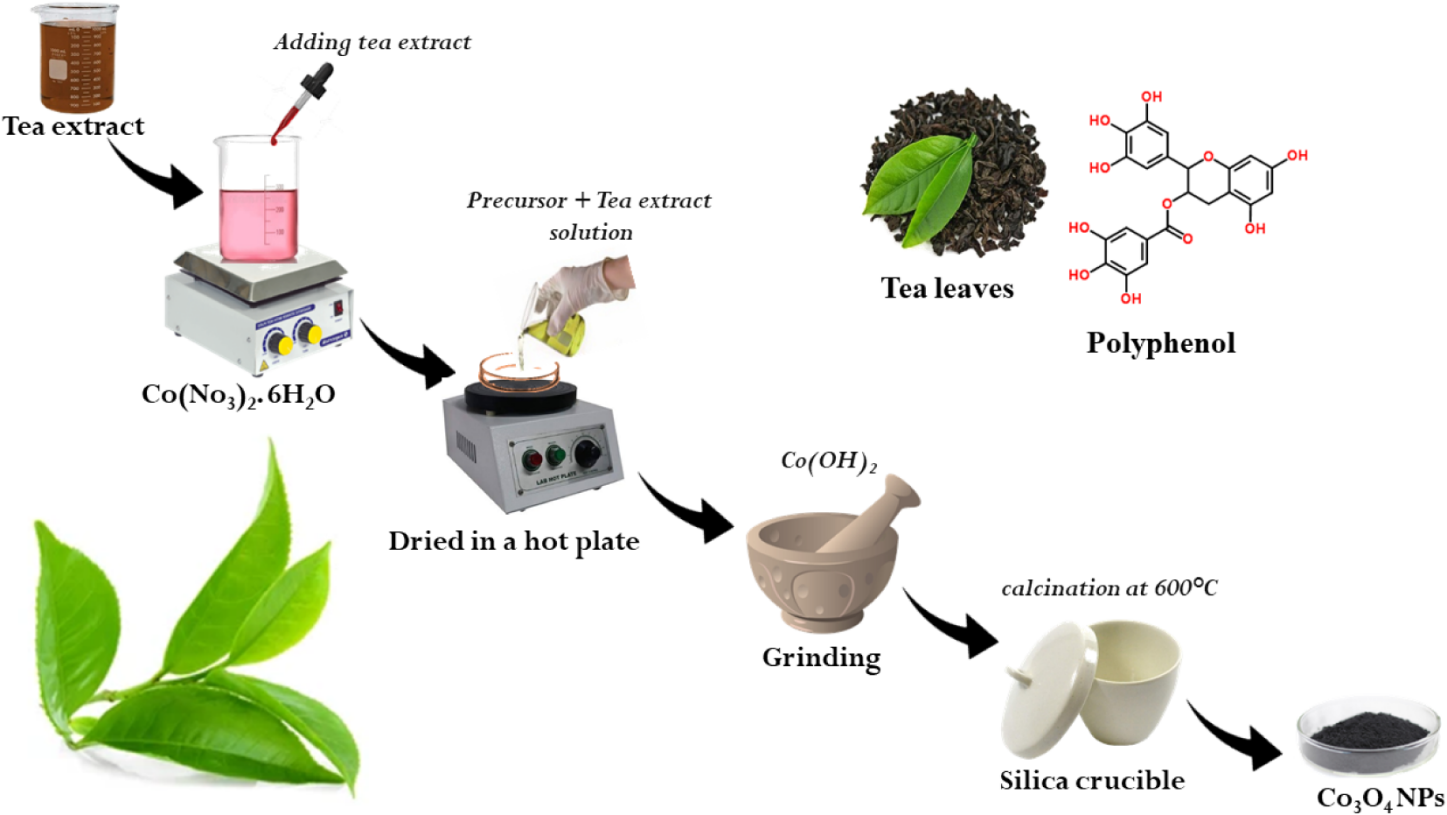
Experimental procedure of the green synthesis of Co_3_O_4_ NS.

### Synthesis of Co_3_O_4_@Gr NCs

Co_3_O_4_ NS composite with graphene by using the following procedure: 0.7 g of the Co_3_O_4_ NS was dispersed in 60 mL of DD water and stirred for 30 min. Simultaneously, 0.2 g of graphene was dispersed in 30 mL of DD water for 30 min. Then, the Co_3_O_4_ NS dispersion was added dropwise to the graphene aqueous dispersion, and the mixture was sonicated for 30 min, followed by stirring for 12 h. The solution was subsequently washed and centrifuged. Finally, the resulting precipitate was dried in an oven at 80 °C for 12 h. Table 1 shows a comparison between the synthesized Co_3_O_4_ NS in this study and those of earlier literature reports.

**Table 1:** Comparison of the As-Synthesized Co_3_O_4_ NS with Earlier Reports

Material Type	Preparation Method	Description	Reference
Pure Co_3_O_4_	Chemical precipitation	Co^2+^ salts are oxidized in alkaline solution, forming Co_3_O_4_ nanoparticles after calcination.	“Synthesis of porous Co_3_O_4_/Reduced graphene oxide by a two-step method for supercapacitors with excellent electrochemical performance.”
	Combustion	Co^2+^ nitrate solution mixed with natural fuels undergoes combustion, yielding Co_3_O_4_ nanoparticles after calcination.	“Cobalt oxide nanoparticles for biological applications: synthesis and physicochemical characteristics for different natural fuels.”
	Coprecipitation	Co_3_O_4_ nanoparticles were synthesized from analytical grade cobalt nitrate and ammonium oxalate via coprecipitation, followed by drying and calcination at 400–500 °C	“Synthesis and characterization of cobalt oxide (Co_3_O_4_) nanoparticles.”
	Sol–gel	Co NPs were synthesized by the sol–gel method using cobalt nitrate with/without capping agents, followed by gel formation, aging, and calcination.	“Efficient photocatalytic degradation of Malachite green dye using facilely synthesized cobalt oxide nanomaterials using citric acid and oleic acid.”
	Microwave-assisted solvothermal method	CoO nanoparticles were synthesized from cobalt(II) aacetylacetonate (Co(acac)_2_) using a microwave-assisted solvothermal method, followed by washing and drying.	“Surfactant-free synthesis and magnetic property evaluation of air-stable cobalt oxide nanostructures.”
	Solvothermal method	Metallic cobalt nanoparticles were synthesized by a solvothermal process using Co(Ac)_2_·4H_2_O and SDBS in ethanol, followed by washing and drying.	“Preparation of cobalt oxide nanoparticles and cobalt powders by solvothermal process and their characterization.”
	Thermal decomposition method	Co_3_O_4_ nanoparticles were obtained by thermal decomposition of [Co(NH_3_)_4_CO_3_]NO_3_·H_2_O precursor at 150–300 °C in air.	“Synthesis, characterization, and investigation of optical and magnetic properties of cobalt oxide (Co_3_O_4_) nanoparticles.”
Co_3_O_4_/Graphene Composite	Hydrothermal	Co^2+^ and graphene oxide mixed, self-assemble during hydrothermal reaction; often followed by reduction of graphene.	“One-step synthesis of Co_3_O_4_ nanoparticles/laser-induced graphene composites in ambient conditions for electrocatalytic OER reaction.”
	Laser-induced one-step synthesis	Cobalt salt-soaked substrate (e.g., wood) subjected to a focused laser, forming Co_3_O_4_ and graphene simultaneously.	“Oxygen-vacancy abundant ultrafine Co_3_O_4_/graphene composites for high-rate supercapacitor electrodes.”
	Chemical deposition/reduction	Co_3_O_4_ nanoparticles are deposited onto graphene oxide sheets, and then the graphene is reduced to rGO.	“Oxygen-vacancy abundant ultrafine Co_3_O_4_/graphene composites for high-rate supercapacitor electrodes.”
	Sol–gel/hybrid	Combination of sol–gel method with graphene dispersion for uniform nanoparticle anchoring.	“Synthesis of porous Co_3_O_4_/Reduced graphene oxide by a two-step method for supercapacitors with excellent electrochemical performance.”

## Results and Discussions

### Structural Properties

The crystalline structures of the as-prepared Co_3_O_4_ and Co_3_O_4_@Gr samples were analyzed by X-ray diffraction. Figure 2A shows the diffraction peak patterns at 2θ of 19.11°, 31.37°, 36.94°, 38.71°, 44.85°, 59.38°, and 65.34°, corresponding to the (111), (220), (311), (222), (400), (511), and (440) planes, respectively.^46–48^ The observed diffraction peaks of the cobalt oxide nanoparticles (NS) can be well indexed to the cubic crystal structure of Co_3_O_4_, as confirmed by JCPDS card no. 42-1467.^9^

**Figure 2:**
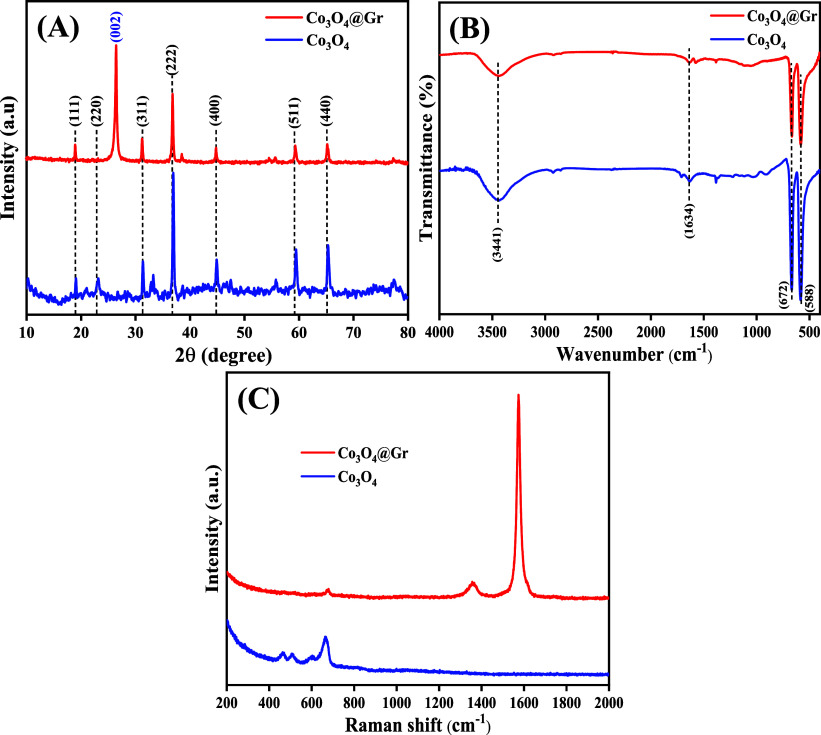
(A) XRD patterns, (B) FT-IR spectra, and (C) Raman spectra of Co_3_O_4_ and Co_3_O_4_@Gr NCs.

The sharpness of the XRD peaks without the presence of other impurity peaks indicates the high crystal purity of the prepared Co_3_O_4_ NS. Using Scherrer’s formula, the crystallite sizes of Co_3_O_4_and Co_3_O_4_@Gr NCs were calculated to be 17.91 and 29.7 nm, respectively. The bond formation between the Co_3_O_4_ and Co_3_O_4_@Gr NCs is further analyzed by using the FT-IR technique, as depicted in Figure 2B. For the as-prepared Co_3_O_4_ NS, the FT-IR spectra were traced in the 4000–400 cm^–1^ region.

The identification of the cubic phase Co_3_O_4_ NS was confirmed by the two distinct and sharp FT-IR peaks emerging at 588 and 672 cm^–1^. These peaks are due to the characteristic bands of Co–O, ascribing to metal oxide rocking vibrations. The vibrations associated with O–H stretching and bending, caused by adsorbed water molecules, are responsible for the two distinct vibrational bands at 3441 and 1634 cm^–1^, respectively.^11,49^ The purity of the Co_3_O_4_ NSs may be further confirmed by the identification of the Co–O band. Additionally, Figure 2C illustrates the Raman spectra of the Co_3_O_4_ and Co_3_O_4_@Gr samples by using 532 nm diode lasers. Co_3_O_4_ exhibits Raman peaks at 465, 510, 617, and 664 cm^–1^, ascribing to the E_g_, F^2^_2g_, F^3^_2g_, and A_1g_ symmetry of the spinel Co_3_O_4_ stretching mode, respectively. While Co_3_O_4_@Gr appears to have the Raman peaks at 1345 and 1570 cm^–1^, corresponding to the D band and G band characteristics, along with the A_1g_ symmetry of spinel Co_3_O_4_ at 666 cm^–1^.^50,51^

### Morphological Analysis

To study the surface morphologies and elemental compositions of Co_3_O_4_ and Co_3_O_4_@Gr NCs, FESEM-EDX with elemental mapping was performed. The representative morphologies of the pristine Co_3_O_4_ NS and Co_3_O_4_@Gr NCs in Figure 3A–L reveal a cluster structure of nanospheres stacking on each other, with an average particle size of 81.40 nm. The Co_3_O_4_@Gr NCs show the same structure as Co_3_O_4_ nanospheres loaded onto a graphene sheet. The presence of Co_3_O_4_ nanospheres on the graphene surface effectively prevents stacking and the aggregation of graphene sheets. EDAX (Figure 3D and H) and mapping images also clearly identify the element distribution of Co_3_O_4_@Gr, consisting of Co, O, and C, respectively, as displayed in Figure 3I–L.

**Figure 3:**
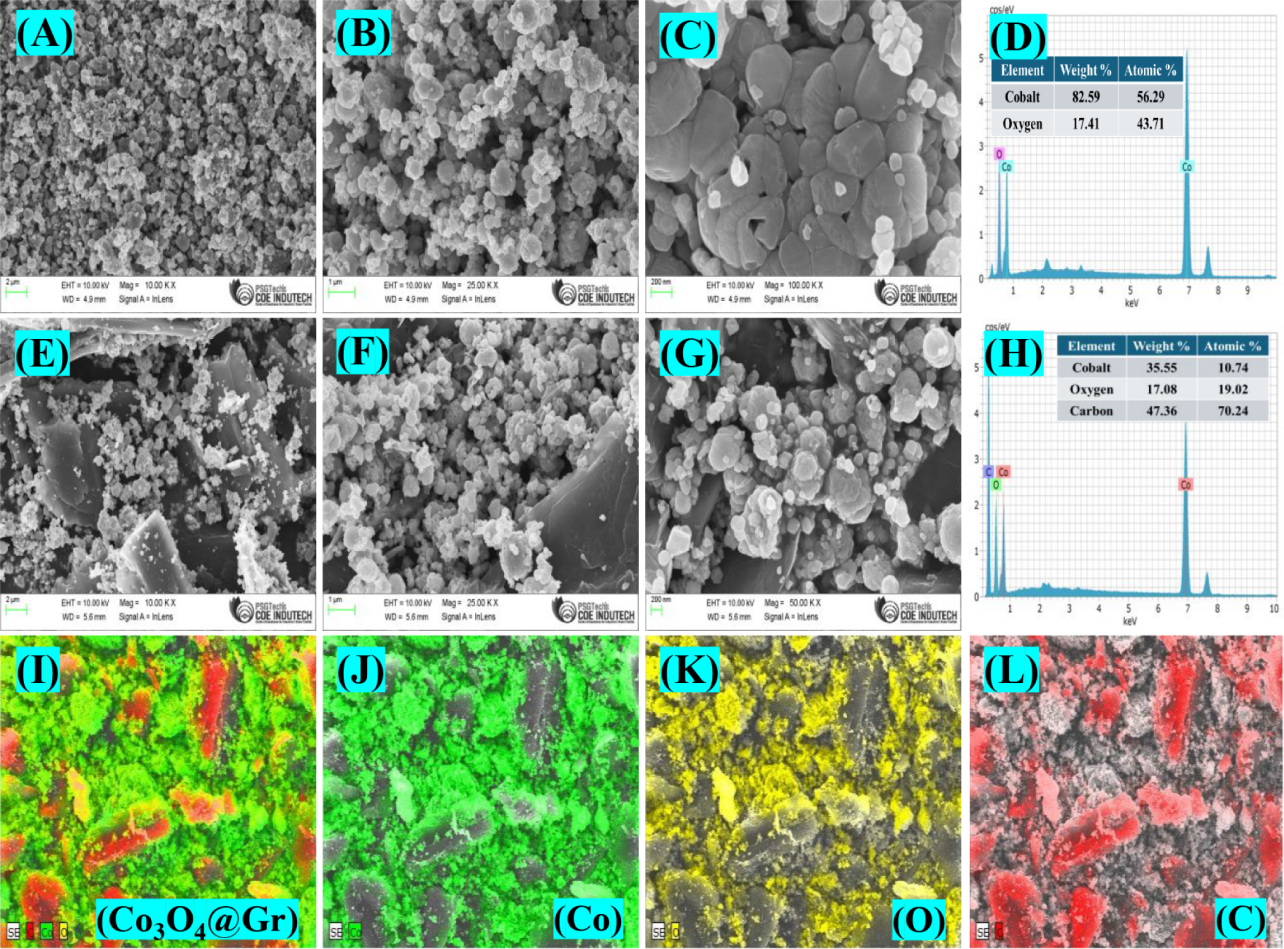
FESEM images of (A–C) Co_3_O_4_, (D) EDAX image of Co_3_O_4_, FESEM images of (E–G) Co_3_O_4_@Gr NCs, (H) EDAX image of Co_3_O_4_@Gr NCs, and Elemental mapping images of (I) Co_3_O_4_@Gr NCs, (J) Co, (K) O, and (L) C.

### Vibrating Sample Magnetometer (VSM) Analysis

Figure 4 represents the M-H hysteresis curve of Co_3_O_4_ NS and Co_3_O_4_@Gr NCs. The shape of the M-H hysteresis loops of the Co_3_O_4_ NS and Co_3_O_4_@Gr NCs indicates weak ferromagnetic behavior. In bulk antiferromagnetic materials, the net magnetization is zero due to the complete compensation of the sublattice magnetizations. From this VSM analysis, the coercivity and remanence magnetization of the as-synthesized Co_3_O_4_ NS were found to be 6.45 Oe and 0.00024 emu/g, respectively. For Co_3_O_4_@Gr NCs, the coercivity and remanence magnetization are 6.45 Oe and 0.00028 emu/g, respectively. The saturation magnetization of Co_3_O_4_@Gr NCs (0.013 emu/g) is marginally higher than that of the Co_3_O_4_ NS (0.0054 emu/g). The weak ferromagnetic nature of Co_3_O_4_ NS can be attributed to the contribution of surface spins or definite size effects. At room temperature, the coercivity values of Co_3_O_4_@Gr NCs are higher than those of the Co_3_O_4_ NS, which indicates the high shape anisotropy of Co_3_O_4_@Gr NCs, which allows them to magnetize all directions along their easy magnetic axis.^49,52^ As a result, the high surface area of Co_3_O_4_@Gr NCs leads to more pronounced surface effects, thereby enhancing the magnetic behavior.

**Figure 4:**
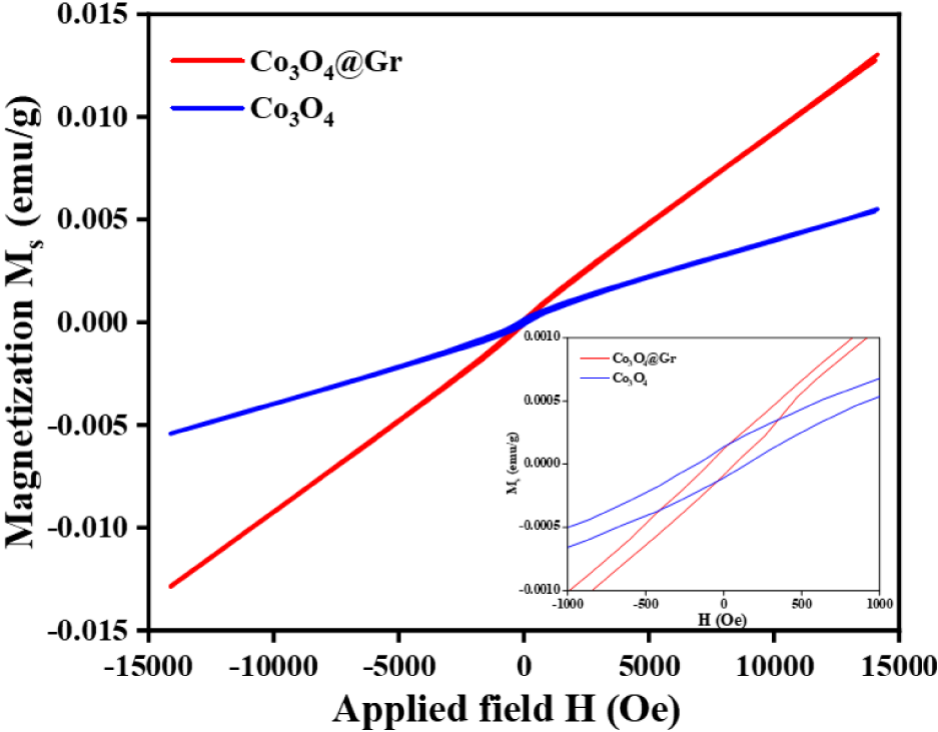
Vibrating Sample Magnetometer analysis of Co_3_O_4_ NS and Co_3_O_4_@Gr NCs.

### Optical Analysis

Figure 5A–C represents the UV–vis spectrum, energy band gap, and photoluminescence (PL) of pristine Co_3_O_4_ NS and Co_3_O_4_@Gr NCs. The as-synthesized Co_3_O_4_ NS and Co_3_O_4_@Gr NCs were dispersed in DI water and sonicated for 15 min. From UV–vis spectroscopy analysis, the absorption peaks of Co_3_O_4_ NS and Co_3_O_4_@Gr NCs were observed at 268 and 266 nm, respectively (Figure 5A).

**Figure 5:**
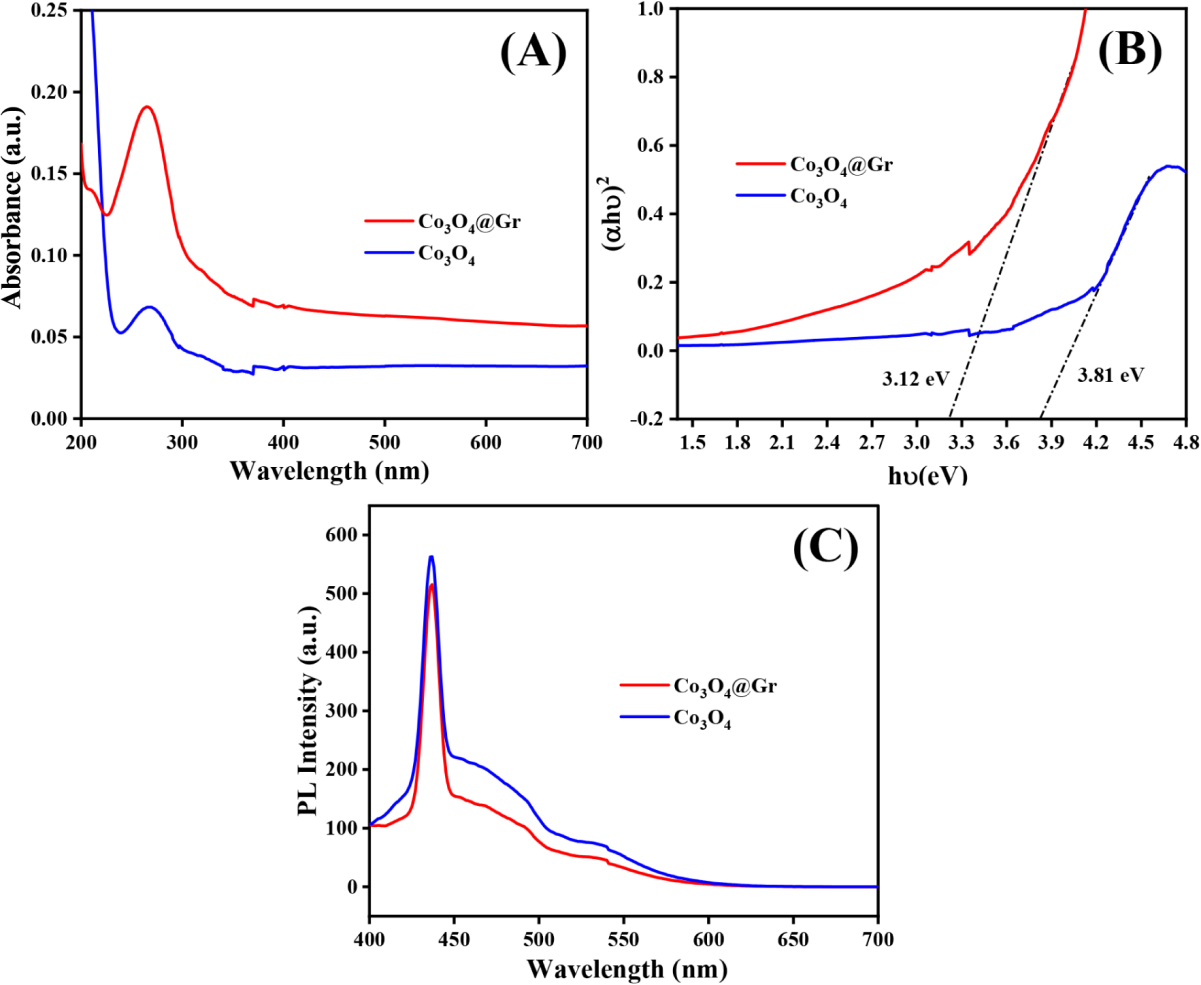
(A) UV–vis spectrum, (B) Tauc’s plot, and (C) PL intensity of Co_3_O_4_ NS and Co_3_O_4_@Gr NCs.

The absorption peak arises predominantly from charge-transfer transitions between O^2–^ ions and Co^2+^/Co^3+^ ions within the intricate crystal structure. This phenomenon entails the excitation of electrons moving from the oxygen 2*p* orbitals to the unoccupied 3d orbitals of Co^2+^/Co^3+^. Moreover, the 3*d* orbital characteristics of Co^2+^/Co^3+^ ions can be enhanced by graphene sheets, resulting in a small shift in the absorption peak. Figure 5B shows the energy band gap of Co_3_O_4_ NS and Co_3_O_4_@Gr NCs, which were calculated using Tauc’s plot and found to be 3.81 and 3.12 eV, respectively. Figure 5C represents the PL spectra of as-synthesized Co_3_O_4_ NS and Co_3_O_4_@Gr NCs, which represent the emission peaks at 436 nm. This emission peak corresponds to the oxygen vacancy, which is associated with the Co_3_O_4_ NS and Co_3_O_4_@Gr NCs, maintaining the cubic structure of the as-synthesized materials. Moreover, the lower intensity of the Co_3_O_4_@gr NCs indicates that the less excited electrons are rebounded to the ground state, which results in the low concentration of charge carriers compared to Co_3_O_4_@Gr NCs.^53–56^

### Photocatalytic Performance and Mechanism

The MB dye-removal activity at a concentration of 1 ppm (1 mg/mL) was evaluated by using the as-prepared NCs. The series of photocatalytic dye-degradation experiments was conducted under sunlight at 9.9759° N and 78.1393° E (Madurai, India). The dye degradation activity experiments were conducted by mixing 10 mg of the photocatalyst and 0.01 g ${\mathrm{Na}}_{2}{\mathrm{SO}}_{3^{•-}}$ (sulfite radical activator) with 150 mL of MB dye and placing it in a dark place without any light source to get the equilibrium of adsorption–desorption. The pH of the solution was adjusted to ∼7 with 30% HCl. Once the dark stirring process was complete, the solution was placed in sunlight to initiate the reaction. The whole experimental setup temperature was maintained at 28–30 °C by adding cold water around the reaction beaker. At 0, 20, 40, 60, 80, and 100 min, a 2 mL aliquot was collected using a syringe and centrifuged at 5000 rpm to remove residual particles. The centrifuged samples were examined using a UV–vis spectrometer at a maximum absorbance wavelength of 662 nm. The percentage of removal efficiency was determined using eq 1:^14,16^

1 $$\mathrm{Degradation efficiency}=\frac{C_{0}-C_{t}}{C_{0}}\times 100$$

where *C*_0_ is the concentration at zero min (before irradiation) and *C_t_* is the concentration at the final time interval (*t* = 100 min).

The photocatalytic dye removal graphs for the bare and NCs materials are presented in Figure 6A–D. The degradation absorbance graph shows that the absorbance peak intensity decreases with an increasing irradiation time, indicating that the dye removal rate increases with an increasing light exposure time. The bare Co_3_O_4_ NS (Figure 6A) exhibits a 45% degradation efficiency at 100 min. The lower efficiency of Co_3_O_4_ NS compared to Co_3_O_4_@Gr NCs can be attributed to their wider band gap, which leads to a faster recombination rate of photoexcited charge carriers. At the same time, the Co_3_O_4_@Gr NCs material exhibits a dye-removal rate of 80% (Figure 6B), approximately 2-fold higher than that of the bare material.

**Figure 6:**
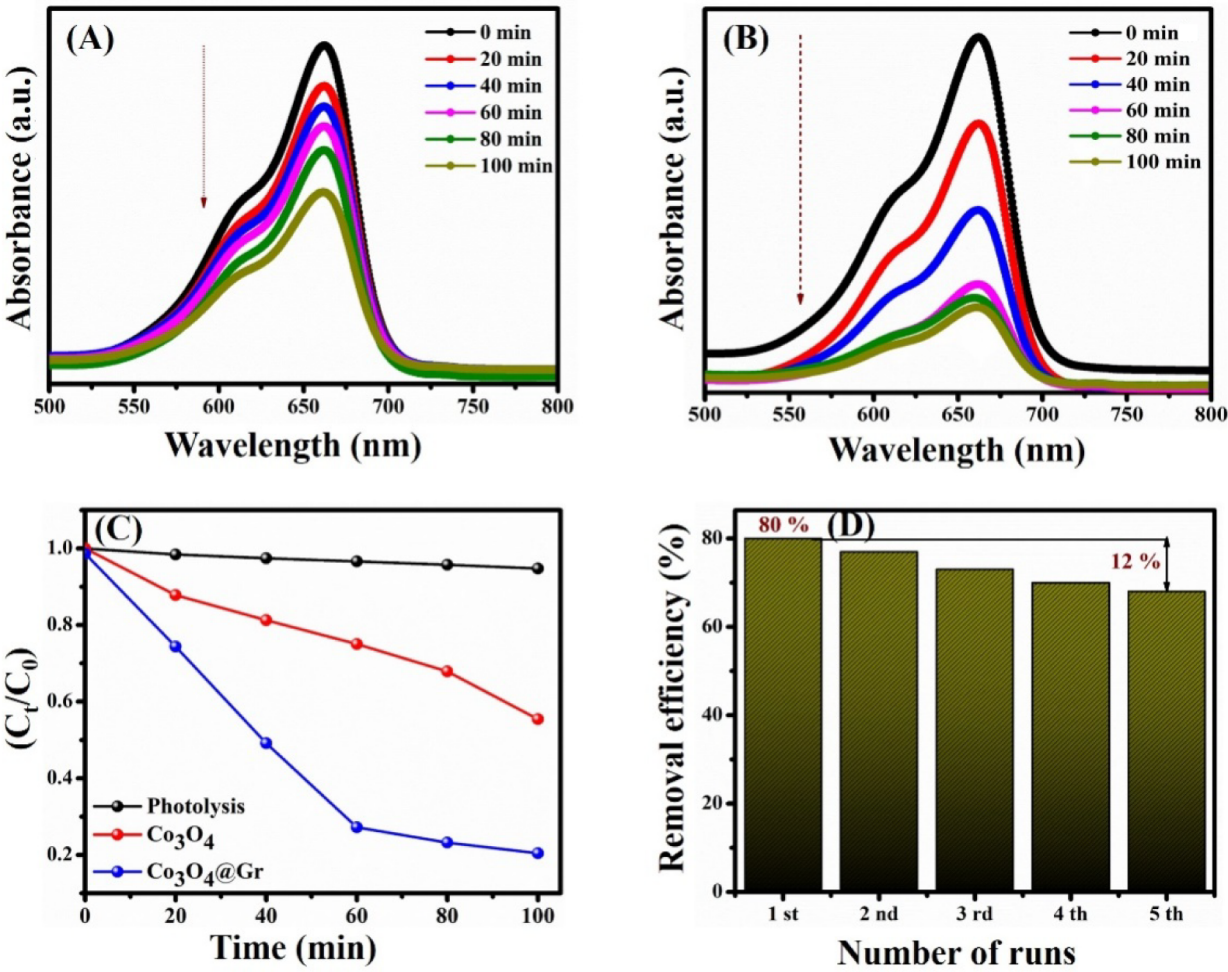
Photocatalytic dye degradation activity of (A) Co_3_O_4_, (B) Co_3_O_4_@Gr NCs, (C) efficiency graph, and (D) reusability graph of Co_3_O_4_@Gr NCs.

The increased efficiency demonstrates that incorporating Gr into bare Co_3_O_4_ enhances the degradation rate, as evidenced by the morphological analysis. The formation of the Co_3_O_4_@Gr composite increases light absorption capacity as well as minimizes the rate of recombination of photogenerated charge carriers. A suitable amount of Gr incorporation formed an effective bond complex within the NCs, thereby increasing the migration of photoexcited electrons via this heterogeneous pathway. Thus, the overall dye-removal activity also increased.^14^ Figure 6C shows the efficiency graph for the NCs, confirming that dye degradation occurs upon the addition of the photocatalyst. In the absence of a photocatalyst, no noticeable change was observed during photolysis.

For an ideal photocatalyst, the photostability is crucial for multiple runs and practical use. Thus, a stability test was carried out under the same conditions as the dye solution: 150 mL; catalyst: 0.012 g; solution concentration: 1 ppm; and light condition: sunlight. After five consecutive cycles, the degradation rate decreased to 68% (i.e., 12% loss), possibly due to catalyst loss during cleaning and centrifugation (Figure 6D). The crystallite size, particle size, band gap, and dye-removal efficiency of the prepared samples are presented in Table 2.

**Table 2:** Comparison of Crystallite Size, Particle Size, Energy Band Gap, and Degradation Efficiency of Co_3_O_4_ NS and Co_3_O_4_@Gr NCs

Samples	Crystallite size (nm)	Particle size (nm)	Band gap (eV)	Efficiency (%)
Co_3_O_4_	17.91	81.40	3.81	45
Co_3_O_4_@Gr	29.7	100.06	3.12	80

When photons with energy equal to or higher than the band gap get absorbed by the NCs, electrons (e^–^) get excited from the valence band to the conduction band, leaving behind positively charged holes (h^+^) in the valence band. Generally, these excited electrons participate in the reduction reaction via the formation of the superoxide radical (O_2_^–^), and the holes in the valence band facilitate oxidation reactions to produce hydroxyl radicals (•OH). The photocatalytic dye removal activity occurs via three steps: (i) the CB-excited e^–^ on the surface of the material further reacts with oxygen (O_2_) to form superoxide radical (O_2_^–^), (ii) direct oxidation of SO_3_^2–^ to SO_3_^–•^ by the holes in the valence band, and (iii) holes in the valence band could react with the water molecules to form •OH.^12,57^ Among the three pathways, the first is not feasible for the degradation of our NC materials because of a potential mismatch between the conduction-band potential of the Co_3_O_4_@Gr NCs and the corresponding redox potential. So, the possibility of generating •OH radicals through photogenerated electrons is not possible in the Co_3_O_4_@Gr NCs. The calculated valence band potential for the Co_3_O_4_@Gr NCs is more positive than the redox potential, indicating that the generation of photoinduced holes in the valence band of Co_3_O_4_@Gr NCs will play a significant role in the degradation of dye via direct oxidation of SO_3_^2–^ to SO_3_^–^, and indirect oxidation of water molecules to •OH. The detailed degradation mechanism of MB using Co_3_O_4_@Gr NCs is schematically depicted in Figure 7.

**Figure 7:**
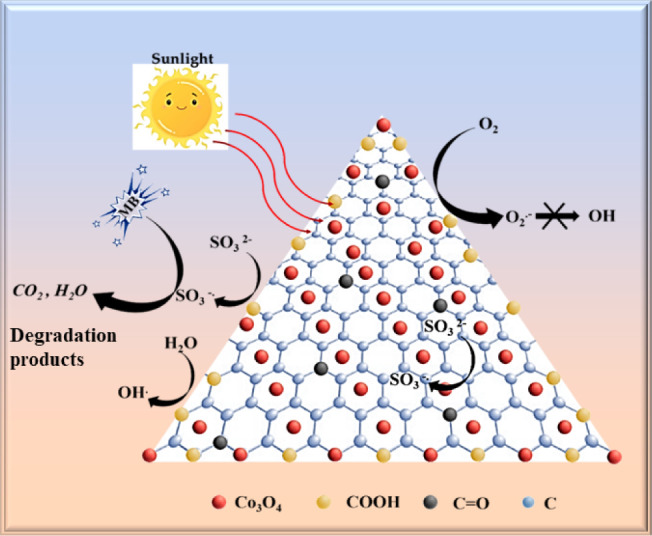
Schematic of the MB dye degradation mechanism by the activation of Na_2_SO_3_^•–^ by Co_3_O_4_@Gr NCs.

Under sunlight irradiation, Co_3_O_4_ NS and Co_3_O_4_@Gr NCs absorb photons with energy greater than or equal to their band gap, generating electron–hole pairs. The photoexcited electrons (e^–^) in the conduction band migrate to the surface and react with dissolved oxygen (O_2_) to form superoxide radicals (•O_2_^–^). These •O_2_^–^ radicals are moderately reactive species capable of attacking methylene blue (MB) molecules, initiating oxidative degradation and ring opening. Simultaneously, photogenerated holes (h^+^) in the valence band oxidize surface hydroxide ions (OH^–^) or water molecules to produce hydroxyl radicals (•OH).

2 $${\mathrm{Co}}_{3}O_{4}+h\nu \to e_{CB}^{-}+h_{VB}^{+}\;\left(\mathrm{Rapid EHPs recombination}\right)$$

3 $${\mathrm{Co}}_{3}O_{4}@\mathrm{Gr}+h\nu \to e_{CB}^{-}+h_{VB}^{+}\;\left(\mathrm{EHPs recombination was suppressed},\;\mathrm{large surface area}\right)$$

4 $$e_{CB}^{-}+O_{2}\to O_{2}^{-}$$

5 $$h_{VB}^{+}+H_{2}O\to •OH$$

6 $$h_{VB}^{+}+OH^{-}\to •OH$$

7 $$•OH+dye\to CO_{2}+Degradation\;products$$

8 $$O_{2}^{-}+dye\to CO_{2}+Degradation\;products$$

The •OH radicals possess a much higher oxidation potential and play a dominant role in mineralizing MB, breaking down intermediate species into CO_2_ and H_2_O. Hence, both hydroxyl and superoxide radicals synergistically contribute to overall dye degradation.

### Electrochemical Detection of Dopamine at Co_3_O_4_@Gr/GCE

The electrochemical detection of dopamine (DPA) at the Co_3_O_4_@Gr/Glassy carbon electrode (GCE) was examined and compared with that at other modified electrodes, including Co_3_O_4_/GCE, Gr/GCE, and bare GCE. The results are displayed in Figure 8A. The Co_3_O_4_@Gr/GCE exhibits a CV response with a distinct oxidation peak for DPA (200 mM DPA) at 0.56 V, attributed to a one-electron transfer process that generates the DPA^•+^ free radical. At 0.65 V, this unstable free radical (DPA^•+^) undergoes additional oxidation, forming the diphenylbenzidine (DPB) dimer. While the DPB was undergoing redox cycling, it exhibited the expected oxidation and reduction peaks. On the other hand, the oxidation response of DPA was recorded using the other modified electrodes, which exhibit comparatively lower oxidation peak currents at higher voltages than those of Co_3_O_4_@Gr/GCE (see the corresponding bar chart in Figure 8B). It reveals that incorporating Co_3_O_4_ over Gr via a strong synergistic interaction can comparatively increase electrocatalytic activity.^42–45^ Finally, Co_3_O_4_@Gr/GCE is a promising electrode material for the electrochemical detection of DPA.

**Figure 8:**
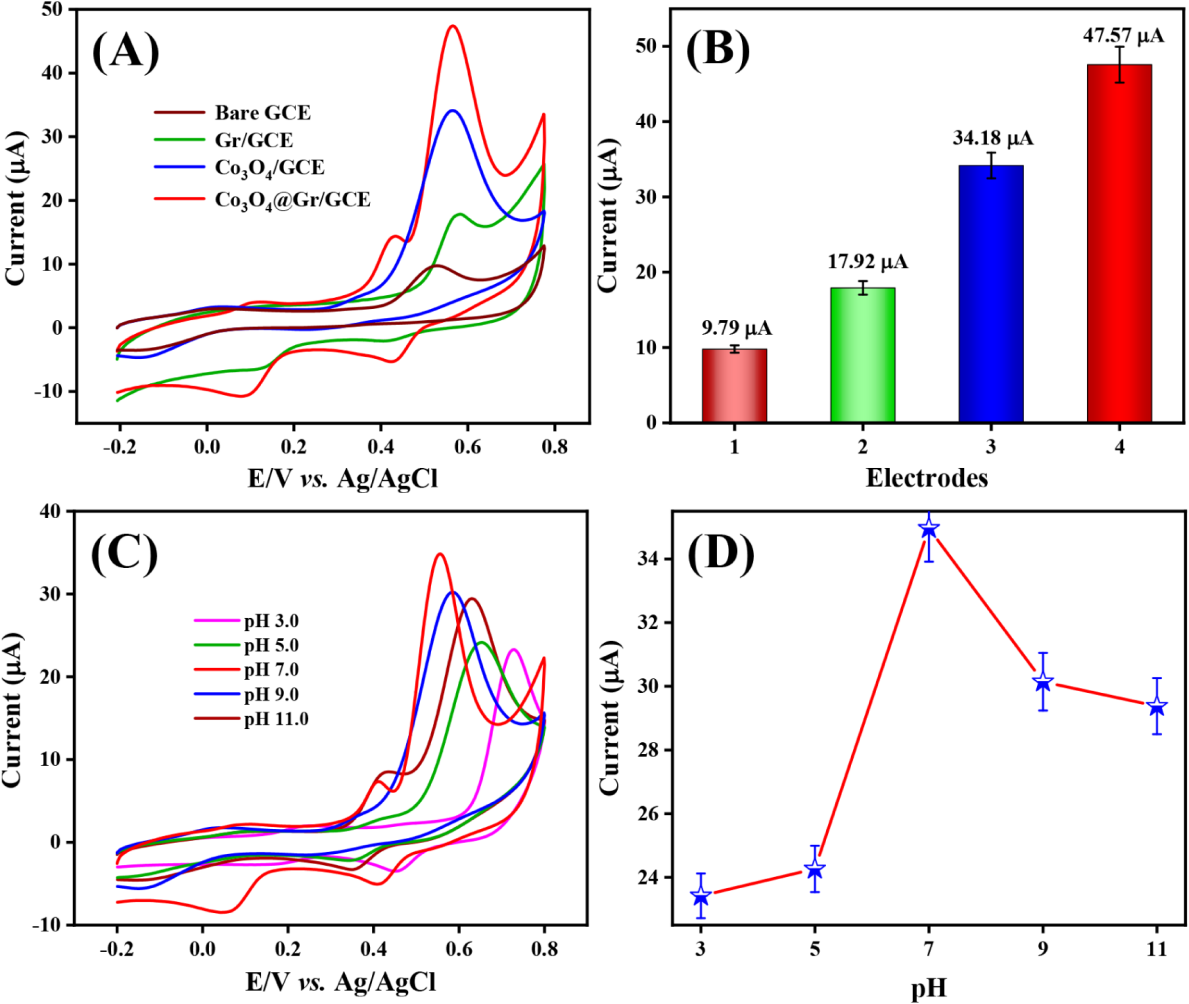
(A) CV responses of bare GCE, Co_3_O_4_/GCE, Gr/GCE, and Co_3_O_4_@Gr/GCE in the presence of 200 mM DPA with 0.05 M PBS (pH 7.0) at a scan rate of 50 mV/s, (B) corresponding bar diagram of oxidation current for DPA over different modified GCEs. (C) CV signals of Co_3_O_4_@Gr/GCE at different pH (3.0–11.0) of 0.05 M PBS with 150 mM DPA at a scan rate of 50 mV/s, (D) the corresponding calibration linear plot.

### Effect of pH

The electrocatalytic performance of the working electrode in the electrochemical sensor is significantly influenced by the pH condition of the supporting electrolyte (modified electrode). The electrocatalytic activity of Co_3_O_4_@Gr/GCE was examined by varying the pH of 0.05 M PBS buffer from 3.0 to 11.0 in the presence of DPA 150 nM. According to the CV signal results shown in Figure 8C, the oxidation peak current of DPA increased rapidly with an increase in pH, reaching 7.0. Subsequently, as the pH continued to rise, the oxidation peak current gradually decreased. This alteration can result from DPA polymerization and dimerization in acidic and alkaline media, respectively. The resulting calibration plot (oxidation peak current vs pH) shown in Figure 8D indicates a higher oxidation peak current at pH 7.0 for the DPA sensor at Co_3_O_4_@Gr/GCE. Therefore, this pH 7.0-optimized condition of 0.05 M PBS was used in all experiments.^35^

### Effect of DPA Concentration

The sensing of DPA at Co_3_O_4_@Gr/GCE was evaluated by varying the concentration of DPA from 50 to 250 mM in a 0.05 M buffer (pH 7.0) at a scan rate of 50 mV/s. The corresponding CV curve in Figure 9A shows that the peak current increases with an increasing DPA concentration. The linear plot of DPA concentration vs oxidation current (peak 1) is shown in Figure 9B, with a linear equation of *y* = 0.2407*x* + 0.4689 and a correlation coefficient of *R* ^2^ = 0.9954. Furthermore, the linear plot for the log of concentration vs log of oxidation current was plotted with a linear regression equation of *y* = 0.9393*x* – 0.4799 and a coefficient of *R* ^2^ = 0.9912 as displayed in Figure 9C. The slope of the plot is 1, indicating that Co_3_O_4_@Gr/GCE follows first-order kinetics in the detection of DPA.

**Figure 9:**
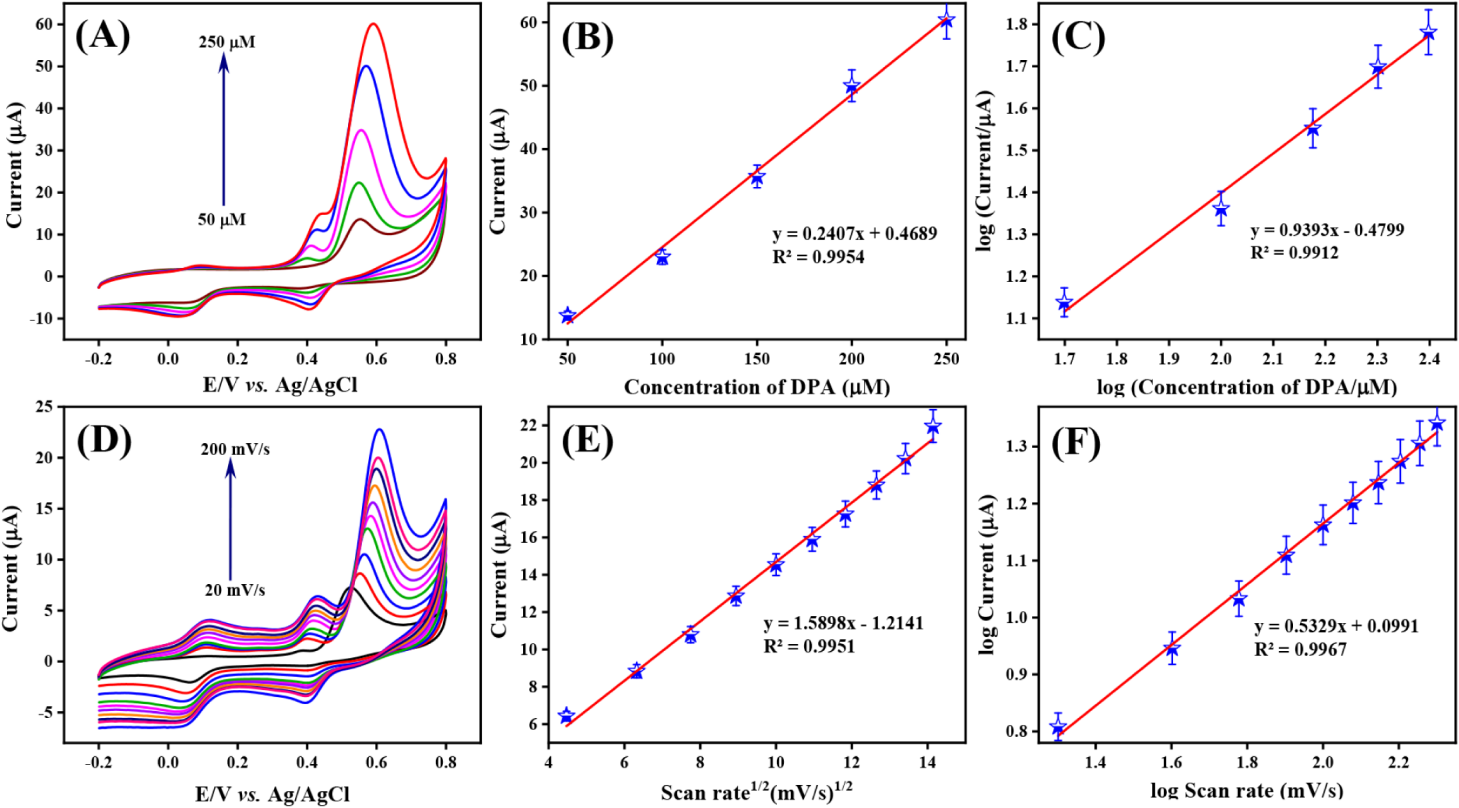
(A) CV response of Co_3_O_4_@Gr/GCE at different concentrations of DPA (50–250 μM) in the presence of 0.05 M PBS (pH 7.0) at a scan rate 50 mV/s, (B) corresponding linear calibration plot of peak current vs concentration of DPA, (C) linear plot for the log of concentration vs log of oxidation current, (D) CV signals at various scan rate (20–200 mV/s) at Co_3_O_4_@Gr/GCE 50 mM DPA in 0.05 M PBS (pH 7.0), (E) the linear plot for oxidation peak current vs square root of scan rate, (F) log current vs log scan rate.

### Effect of Scan Rate

The scan rate is one of the most important factors that influence the electrochemical behavior of modified electrodes. Hence, the effect of scan rate on Co_3_O_4_@Gr/GCE was investigated by varying the scan rate from 20 to 200 mV/s in 0.05 M PBS (pH 7.0) containing 50 mM DPA. The curve displayed in Figure 9D signifies that the oxidation current increases with increasing scan rate. The corresponding linear calibration plot for oxidation peak current vs the square root of scan rate is shown in Figure 9E with a linear regression equation of *y* = 1.5898*x* – 1.2141 and a correlation coefficient of *R* ^2^ = 0.9951. The resulting linear plot of the log of the oxidation peak current vs the log of the scan rate is shown in Figure 9F. From this plot, the corresponding linear regression equation was estimated as *y* = 0.5329*x* + 0.0991, with a coefficient of determination (*R* ^2^) of 0.9967. These results indicate that the overall electrochemical reaction at the DPA sensor on Co3O4@Gr/GCE is surface-controlled.

### Electrochemical Determination of DPA

Differential pulse voltammetry is an appropriate analytical technique for understanding the electrocatalytic behavior of Co_3_O_4_@Gr/GCE in the determination of DPA. Figure 10A shows the DPVs in 0.05 M PBS over the potential range of −0.1 to 0.9 V in the presence of DPA at various concentrations (0.01 to 224 μM). As shown in Figure 10A, the oxidation peak currents of DPA were linearly increased from 0.01 to 224 μM. The linear relationship between the concentration of DPA and the obtained oxidation peak current was identified using a calibration plot as given in Figure 10B. The calibration plot displays a wide linear response for the range of 0.01 to 224 μM, with a linear regression equation and correlation coefficient of *y* = 0.0274*x* + 1.3794 and *R* ^2^ = 0.9967.^58,59^

9 LOD=3σ/S

10 Sensitivity=Slope/electrode active surface area

**Figure 10:**
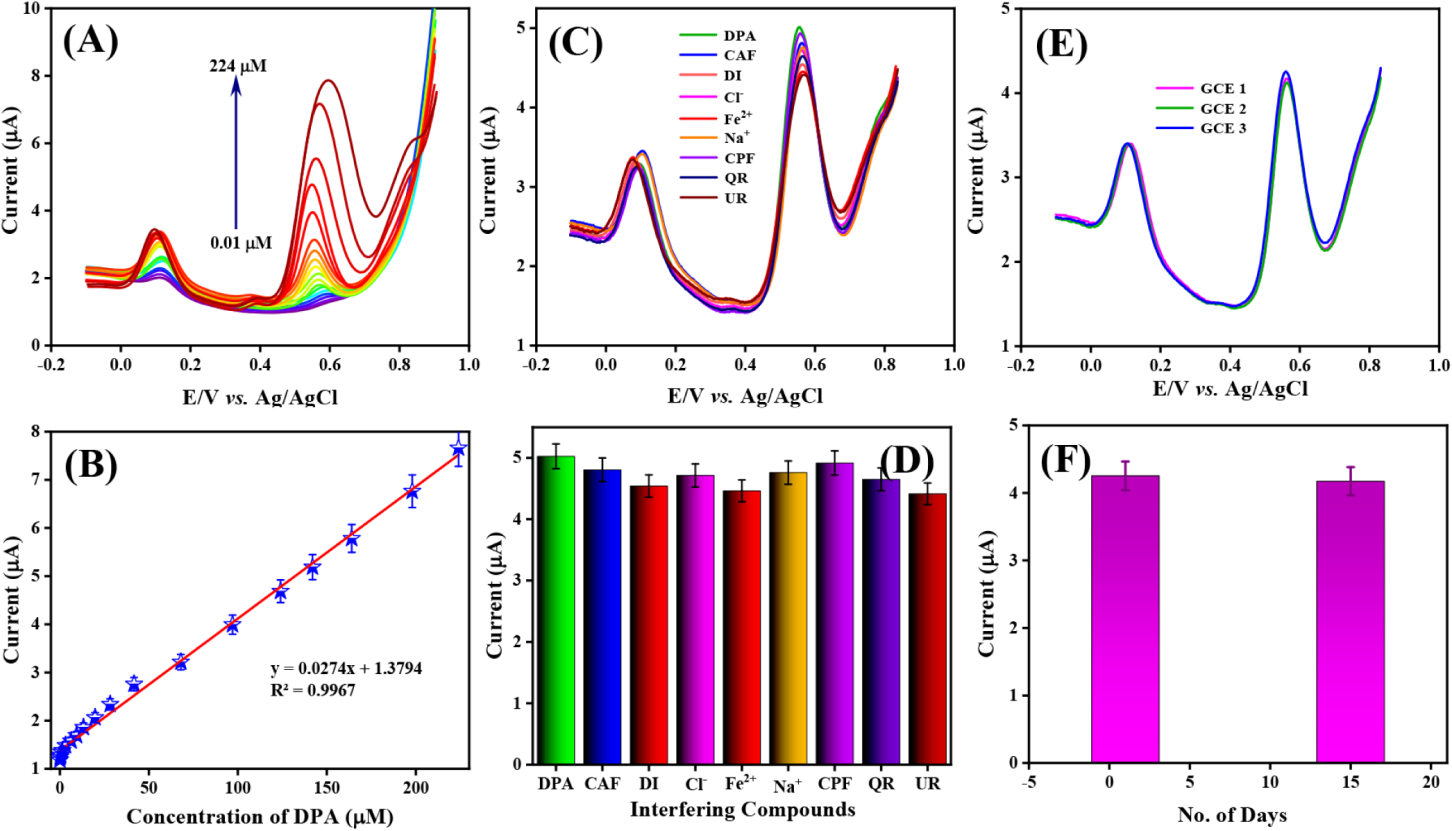
(A) DPV responses of DPA at Co_3_O_4_@Gr/GCE with various concentration ranges from 0.01 to 224 μM containing 0.05 M PBS (pH 7.0), (B) the calibration plot for oxidation peak current vs DPA concentration, (C) interference studies at Co_3_O_4_@Gr/GCE, (D) the corresponding bar diagrams, (E) DPV response for reproducibility studies, (F) bar diagram for storage stability test of DPA at Co_3_O_4_@Gr/GCE.

From the linear plot obtained using the above equations, the lowest detection limit and sensitivity of Co_3_O_4_@Gr/GCE were determined to be 0.006 mM and 2.481 μA μM^–1^ cm^–2^, respectively. These results show that as-prepared Co_3_O_4_@Gr/GCE exhibits superior catalytic activity for DPA detection, with a very low detection limit, a wide linear response, and acceptable sensitivity. Table 3 compares the DPA detection limits of Co_3_O_4_@Gr NCs with those reported previously.

**Table 3:** Comparison of the DPA Detection Limit of As-Synthesized Co_3_O_4_@Gr NCs with Previous Reports

Modified electrode	Technique	Linear range (μM)	LOD (μM)	ref.
CNWO-PPY	DPV	0.01–861	0.0054	^42^
NSCNT-0.5	DPV	5–50	0.09	^58^
N-ZWR-1	DPV	0.2–841	0.0083	^69^
DyCo-LDH/NG	DPV	0.05–470	0.012	^60^
AuNP/Ni–Fe LDH/BPNSs	DPV	0.0125–1003.82	0.00463	^61^
SnMnS@Cu-BDC MOF	DPV	0.4 −220.0	0.78	^62^
CeVO_4_@g-C_3_N_4_	DPV	0.01–792	0.0011	^63^
CF-LS/SnS	DPV	0.0125–803.825	0.0044	^64^
NiFe-LDH	DPV	0.005–226.26	0.001	^65^
**Co**_**3**_ **O**_**4**_ **@Gr/GCE**	**DPV**	**0.01 to 224**	**0.006**	**This work**

### Selectivity, Reproducibility, and Stability toward DPA Detection

The interference, reproducibility, and stability are significant factors for electrochemical sensors. To examine the selectivity of the DPA molecules on Co_3_O_4_@Gr/GCE, we have performed the DPV analysis with 150 μM DPA in the presence of 100 folds’ higher concentration of various foreign compounds such as carbofuran (CAF), diuron (DI), chlorine (Cl^–^), iron (Fe^2+^), sodium (Na^+^), chlorpyrifos (CPF), quercetin (QR), and urea (UR). From Figure 10C, the DPA oxidation peak current response in the presence of interfering compounds was calculated to be 99.7%, 97.2%, 96.0%, 91.5%, and 90.8%, respectively. Herein, the DPA oxidation signals were interfered with below 10%, which implies that the electrochemical detection of DPA on Co_3_O_4_@Gr/GCE is not interfered with by other species, as shown in Figure 10D. Thus, we have confirmed that Co_3_O_4_@Gr/GCE exhibits excellent anti-interference stability for DPA detection.^66,67^ The reproducibility of Co_3_O_4_@Gr/GCE was assessed using three individual Co_3_O_4_@Gr/GCE electrodes and by reporting their current responses to DPA (150 μM) in 0.05 M buffer (pH 7.0). The DPV responses obtained for the oxidation current responses vs the number of Co_3_O_4_@Gr/GCE electrodes are displayed in Figure 10E. Furthermore, the storage stability of the reported sensor, Co_3_O_4_@Gr/GCE, was evaluated using the same experimental procedures followed for reproducibility, in the presence of DPA (150 μM). In this study, the current response of DPA was measured over a 10-day period. Fortunately, Co_3_O_4_@Gr/GCE retains 96.3% of its initial oxidation peak current response after storage, as displayed in Figure 10F. These electrochemical experiments reveal that the reported Co_3_O_4_@Gr/GCE exhibits higher selectivity, stability, and excellent reproducibility for DPA sensing, and thus is promising for practical applications.^68–70^

### Supercapacitor Application

Electrochemical studies, such as cyclic voltammetry (CV), galvanostatic charge–discharge (GCD), and electrochemical impedance spectroscopy (EIS), were carried out through a three-electrode setup (CHI6008e, USA), consisting of the active material (Co_3_O_4_ and Co_3_O_4_@Gr), Ag/AgCl, and platinum wire as the working, reference, and counter electrodes, respectively. To prepare the working electrode, nickel (Ni) foam was used as the substrate (11 cm^2^). A slurry was prepared by mixing the active material, poly­(vinylidene difluoride) (PVDF) as the binder, and mesoporous carbon in the ratio 85:05:10, with an appropriate amount of ethanol as the solvent. The obtained slurry was precisely coated on the Ni foam and dried in a vacuum oven at 80 °C for 4 h before analyzing the electrochemical experiments.

The specific capacitance value can be calculated from the CV analysis using the following equation:^13,14,18^

Csp=∫idvs×ΔV×mFg

The specific capacitance can be calculated from the GCD analysis using the following equation.^13,14,18^

Csp=I×Δtm×ΔVFg

The electrochemical characteristics of Co_3_O_4_ and Co_3_O_4_@Gr were assessed through a comparative CV study, as illustrated in Figure 11A–C. Principally, the area under the CV curve is commonly used to determine the electrochemically active surface area, which indicates the number of electroactive sites participating in redox reactions and the efficiency of electroabsorption between the active surface materials and ions/charges in the electrolyte. The CV curve of the bare Co_3_O_4_ shows a symmetric rectangular shape with a small pair of redox peaks, indicative of good electrical double-layer capacitance behavior and high reversible pseudocapacitance. An anodic peak at −0.1 V and a cathodic peak at 0.5 V indicate the reversible faradaic redox reaction of Co_3_O_4_. Obviously, loading Co_3_O_4_ onto the graphene surface influenced the CV area, enhancing the electrical capacitive characteristics due to the synergistic effect of the double-layer and pseudocapacitance properties of Co_3_O_4_ and graphene. Figure 11B and C displays the CV curves of Co_3_O_4_ and Co_3_O_4_@Gr at scan rates ranging from 10 to 100 mV/s, respectively. The deterioration of the CV curve’s rectangularity is observed in both samples with increasing scan rates, suggesting electrochemical irreversibility and internal electrical resistance (*iR* drop) at higher rates. However, Co_3_O_4_@Gr exhibits higher response current densities and less skewed CV behavior than Co_3_O_4_, indicating better rate capability at high current densities. The comparative applied voltage–time profiles for the galvanic charging/discharging (GCD) curves at 1 A/g for Co_3_O_4_ and Co_3_O_4_@Gr are also examined, as exemplified in Figure 11D–F. Introducing graphene into Co_3_O_4_ significantly increases the discharge time and reduces the potential drop (*iR* drop), suggesting improved electrochemical capacitance behavior. The specific capacitance (*C*_sp_) of Co_3_O_4_ and Co_3_O_4_@Gr calculated from CV and GCD results at various rates is illustrated in Figure 11G and H, respectively. The pristine Co_3_O_4_ NS exhibits specific capacitance of 300 F/g at 5 mV/s and 130 F/g at 1 A/g. Meanwhile, the Co_3_O_4_@Gr electrode undoubtedly exhibits superior rate performance than the bare Co_3_O_4_. The highest specific capacitance values of Co_3_O_4_@Gr, calculated from CV and GCD results, are 349 F/g at a scan rate of 5 mV/s and 158 F/g at a current density of 1 A/g, respectively.

**Figure 11:**
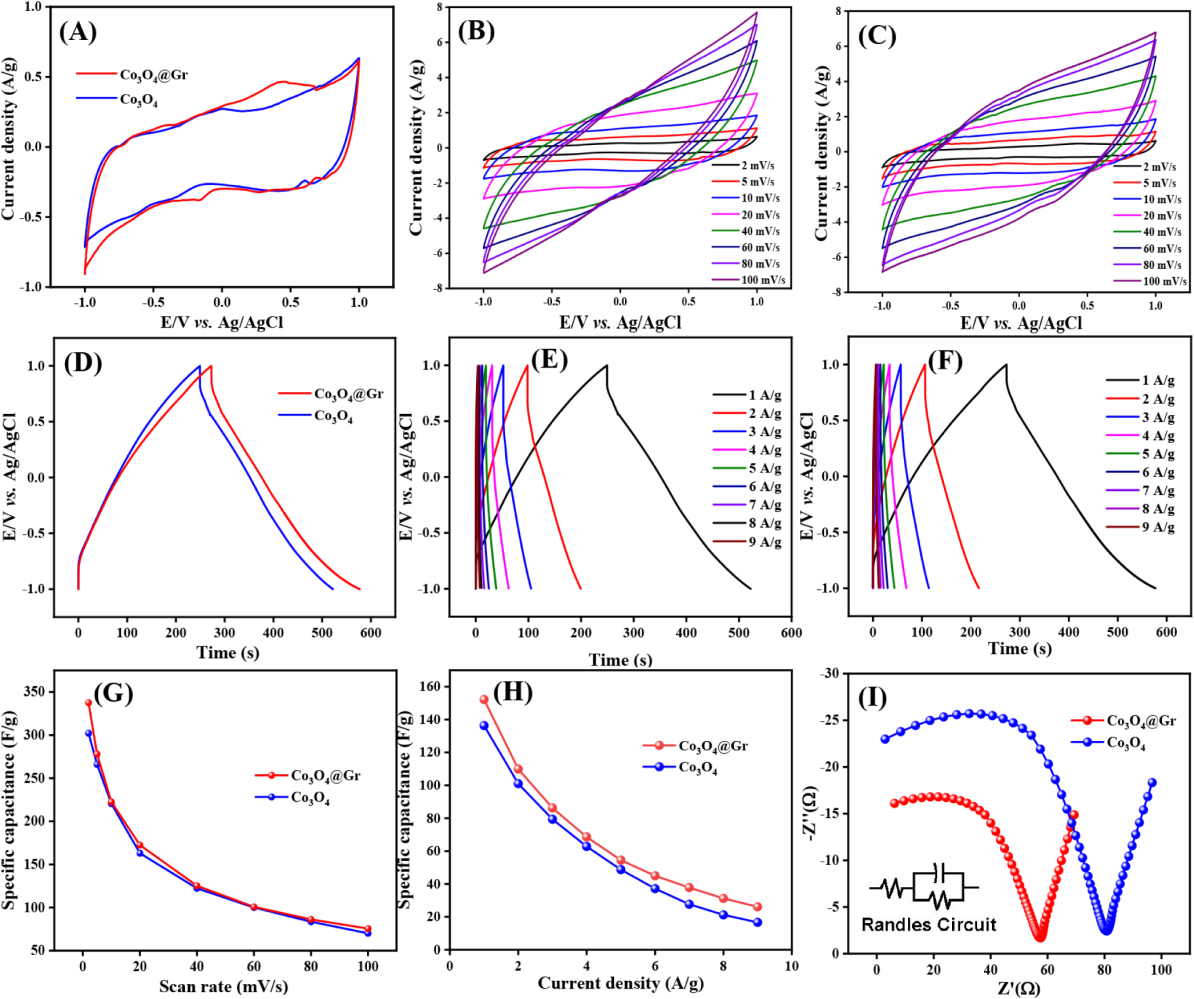
(A) CV curve of theCo_3_O_4_@Gr binary NCs compared with bare Co_3_O_4_ at a scan rate of 2 mV/s, (B) Co_3_O_4_ at different scan rates from 2 to 100 mV/s, (C) Co_3_O_4_@Gr NCs at different scan rates from 2 to 100 mV/s, (D) GCD curve of theCo_3_O_4_@Gr binary NCs compared with bare Co_3_O_4_ at a current density of 1 A/g under the voltage range from −1.0 to 1.0, (E) Co_3_O_4_@Gr, (F) Co_3_O_4_@Gr NCs at different current densities from 1 to 9 A/g, specific capacitance at different (G) scan rates, (H) current densities, and (I) EIS Nyquist plots ofCo_3_O_4_ and Co_3_O_4_@Gr NCs electrodes (inset: The electrical equivalent circuit of Co_3_O_4_@Gr NCs).

The comparative Nyquist plots of Co_3_O_4_ and Co_3_O_4_@Gr using electrochemical impedance spectroscopy (EIS) at a frequency range of 0.01–10,000 Hz are examined, as depicted in Figure 11I. Essentially, the intersection of the initial line with the semicircle’s amplitude corresponds to the overall electrode resistance, associated with electron-transfer-limited processes. A phase angle within the linear region reflects ion/charge diffusion of the materials. Clearly, Co_3_O_4_@Gr exhibits lower electronic/ionic resistances at higher frequencies and a smaller semicircle in the Nyquist plot than bare Co_3_O_4_, indicating faster ion/charge diffusion and lower total electrode resistance. The solution resistance (*R*_s_) and the charge transfer resistance (*R*_ct_) are determined from the EIS spectra to be 80.2 Ω and 53 Ω, respectively. This lower resistance indicates that the graphene layers enhance ionic conductivity and shorten the diffusion path, thereby improving electrochemical performance. This result confirms that the electrical conductivity and ion-charge transport in Co_3_O_4_@Gr are improved, attributed to the synergistic effect of Co_3_O_4_ NS loading on the graphene surface.

## Conclusions

This work demonstrates the efficient synthesis of Co_3_O_4_ NS and Co_3_O_4_@Gr NCs via microwave-assisted and solution-mixing methods using green tea leaf extract as the reducing agent. The Co_3_O_4_@Gr NCs exhibit a cubic spinel crystal structure with high crystallinity, where the Co_3_O_4_ NS is spherically agglomerated and uniformly distributed on the graphene surface. The Co_3_O_4_@Gr NCs show a significant suppression of charge carrier recombination between hybrid orbitals within the composite structure. These NCs achieve 80% degradation efficiency against MB dye, outperforming pristine Co_3_O_4_, which exhibits only 45% efficiency. This improvement is attributed to graphene’s role in inhibiting electron–hole recombination between the valence and conduction bands. The proposed sensor based on Co_3_O_4_@Gr shows a wide linear response range of 0.01 to 224 μM, an impressively low detection limit for DPA (0.006 μM), and an excellent sensitivity (2.481 μA μM^–1^ cm^–2^). The Co_3_O_4_@Gr-modified GCE also demonstrates remarkable selectivity for various pesticides, fungicides, and metal ions alongside outstanding reproducibility and stability. Electrochemical studies reveal the highest specific capacitance for Co_3_O_4_@Gr, with values of 349 F/g from CV and 158 F/g from GCD at a scan rate of 5 mV/s and a current density of 1 A/g, respectively. Additionally, the lower resistance of Co_3_O_4_@Gr NCs enhances their electrochemical performance, making them suitable for potential future applications.
